# Passive debris cloaking in beetles provides non-visual camouflage against predatory ants

**DOI:** 10.1093/beheco/araf064

**Published:** 2025-07-28

**Authors:** K Greig, T R Buckley, R A B Leschen, G I Holwell

**Affiliations:** Manaaki Whenua Landcare Research, 231 Morrin Road, St. Johns, Auckland 1072, New Zealand; School of Biological Sciences, University of Auckland, Private Bag 92019, Auckland 1010, New Zealand; Manaaki Whenua Landcare Research, 231 Morrin Road, St. Johns, Auckland 1072, New Zealand; Manaaki Whenua Landcare Research, 231 Morrin Road, St. Johns, Auckland 1072, New Zealand; School of Biological Sciences, University of Auckland, Private Bag 92019, Auckland 1010, New Zealand

**Keywords:** anti-predator adaptations, camouflage, chemical crypsis, decorating behaviors, non-visual crypsis, passive debris cloaking

## Abstract

Our understanding of visual camouflage has increased dramatically in recent years, however we know less about anti-predator defenses that exploit senses other than vision. Low light habitats, such as leaf litter, are more commonly dominated by predators that rely on chemical, tactile, and other nonvisual cues. Passive debris cloaking is a trait found in several arthropod groups that reside in low light habitats and appears as a layer of environmental debris that covers the cuticle. This debris accumulates passively as the organism moves through its habitat, generally via the secretion of adhesive compounds through specialized pores. We hypothesized that passive debris cloaking is a form of non-visual camouflage, and tested this experimentally using zopherid beetles as a model. Zopherid beetles are highly diverse in Aotearoa New Zealand and include many species that exhibit passive debris cloaking. By exposing zopherids with varying degrees of cuticular debris to colonies of foraging predatory ants, we found that passive debris cloaking (1) reduces detection by ants, (2) reduces the probability of attack if detected, and (3) is most effective when interactions occur on natural backgrounds. Our results provide evidence that passive debris cloaking is a highly effective form of non-visual camouflage, suggesting non-visual camouflage may be more prevalent in low light habitats than currently appreciated.

## Teaser

Some small invertebrates found in leaf litter use passive debris cloaking for concealment. This involves the gradual development of thick debris layers on an insect’s cuticle as it moves through its habitat, found abundantly in zopherid beetles in New Zealand. Here we show that passive debris cloaking reduces detection and attack by predatory ants that rely on senses other than vision to hunt. This highlights the importance of non-visual camouflage in low light systems.

## Introduction

Camouflage is used by diverse prey species to avoid detection and identification by predators. Darwin’s (Darwin 1964) initial observations of camouflage inspired early works from Thayer (1909) and Cott (1940) whose influence in the early 20^th^ century acted as catalysts for camouflage research. More recent research has identified different camouflage strategies including background matching (Merilaita and Stevens 2011; Murali et al. 2021), and disruptive colouration (Cuthill et al. 2005; Endler 2006; Stevens et al. 2009), which both reduce detection, and masquerade (Skelhorn et al. 2010b), which hinders identification of prey. Background matching (Merilaita and Lind 2005), where a prey’s colors and patterns resemble their background and prevent detection by predators, has received the most attention. Sand color matching in tiger beetles (Yamamoto and Sota 2020) and pattern matching in the Fowler’s toad (Barnett et al. 2023) are just two of many examples of animals that match specific components of their habitats to achieve background matching. Disruptive colouration can also hinder detection (Cuthill et al. 2005; Merilaita and Lind 2005), but this is achieved by the creation of false outlines when a prey’s colors and patterns include contrasting markings that extend to the edges of the body. Disruptive colouration can work in conjunction with background matching, such as in bark resting moths *Hypomecis roboraria* and *Jankowskia fuscaria* (Kang et al. 2015), or independently of it (Schaefer and Stobbe 2006). While these strategies influence receiver perception, recent attention has also focused on the cognitive processes of the receiver, and how these can be exploited by camouflaged prey. Masquerade is such a strategy as it influences recognition rather than perception. In an experiment using domestic chicks as predators of twig mimicking caterpillars, it was demonstrated that chicks with prior experience with twigs took longer to identify and attack caterpillars than those with no prior experience (Skelhorn et al. 2010b). This demonstrated the important role of receiver psychology in the evolution of camouflage strategies. Camouflage research has to date largely focused on colors, visual patterns and predominantly visual predators. However, many predators rely on multiple highly developed senses to locate prey.

The use of non-visual cues by predators raises the potential for animals to use non-visual camouflage or other techniques to avoid detection. Indeed, a number of species employ non-visual crypsis to avoid predatory attention, meaning any form of crypsis other than vision (reviewed in (Ruxton 2009)). There are many animals who conceal themselves by matching the olfactory components of their backgrounds—commonly referred to as chemical crypsis, a subset of non-visual crypsis. This occurs in the wooly alder aphid *Chrysopa slossonae* (Eisner et al. 1978), the coral feeding reef fish *Oxymonacanthus longirostris* (Brooker et al. 2015), the puff adder *Bitis arietans* (Miller et al. 2015) and *Mechanitis polymnia* and *Biston robustum* caterpillars (Akino et al. 2004; Henrique et al. 2005). Despite these examples, non-visual camouflage is significantly less well understood than visual camouflage.

One adaptation which may be an overlooked form of non-visual camouflage animals is the accumulation of debris particles from the environment on an animal’s body. A diverse range of small litter dwelling invertebrates accumulate environmental debris over their surface. Debris accumulates onto the cuticle naturally as the animal moves through its environment, usually through the help of modified setae and/or glandular secretions. It typically appears as a layer of debris or soil granules and has been observed in harvestmen (Wolff and Gorb 2016; Porto and Pérez-González 2020), flat bugs (Larivière and Larochelle 2022), mites (Mahunka 1994; Olszanowski 1996; Łochyńska 2008a, 2008b; Bromberek and Olszanowski 2012) and several beetle families (Leschen et al. 2005, 2016). This trait has been described using varied terminology, but here we use the term “passive debris cloaking.”

The beetle family Zopheridae is cosmopolitan in distribution, but is unusually diverse and the fourth largest beetle family in Aotearoa New Zealand (Lord and Leschen 2014; Buckley et al. 2020). The Zopheridae are all saproxylic, most feeding on fungi and are typically found in the woody debris of multiple plant species (Marske et al. 2011). Passive debris cloaking is widespread throughout the family, but shows great variability. Some species are entirely covered in a thick layer of debris, others have a sparse and uneven coating, and some have none, making them an ideal group to explore the function of passive debris cloaking. Using nocturnally foraging ants as predators, zopherid prey with varying levels of passive debris cloaking and natural and neutral backgrounds, we experimentally test the hypothesis that passive debris cloaking functions as non-visual camouflage in zopherids. To do this, we test the following predictions; (1) Beetles with a higher degree of passive debris cloaking will have a lower likelihood of detection by ants, (2) If detected by ants, zopherids with a high degree of passive debris cloaking will be less likely to be recognized as prey and attacked, and (3) Detection and attack by ants will be less likely when beetles are on natural backgrounds.

## Methods

### Ethical note

We acknowledge that we used all the knowledge and tools at our disposal to minimize harm, and provide food, water and appropriate housing to all invertebrates used in these experiments for the duration of the study. On completion of the experiments, we euthanised all invertebrates humanely by putting them in a freezer for 12 h. For detailed care and husbandry information see supplemental material.

#### Collection and maintenance.

We collected zopherid beetles from 8 different species: *Pristoderus bakewelli, Pristoderus insignis, Tarphiomimus indentatus, Epistranus c. f. lawsoni, Protarphius sp. 1, Chorasus sp. 1, Pycnomerus sp. 1, and Rytinotus squamulosus* (n = 110) from various localities around Aotearoa New Zealand to be used as prey. We used ants as predators and collected them as entire colonies from local Auckland reserves, and housed them under laboratory conditions. These consisted of two *Austroponera castanea colonies and two Amblyopone australis colonies* (Ward 2005). For detailed collection and husbandry information see electronic supplemental material.

#### Behavioural experiments.

Initially, we had planned to experimentally remove the debris cloak from one of the more heavily coated species in order to assess the response of predators to individuals of the same species with and without the debris cloak. This had previously been achieved for active debris cloakers, whereby debris can be experimentally, and easily removed (Nakahira and Arakawa 2006). However, in pilot trials preceding our experiments, we attempted multiple methods to remove the debris from the beetle’s cuticle with no success (refer to supplemental material for details). For this reason we instead broadened our study to eight species (listed above) varying naturally in their degree of debris cloaking, using a comparative approach rather than experimental removal.

**Fig. 1.**
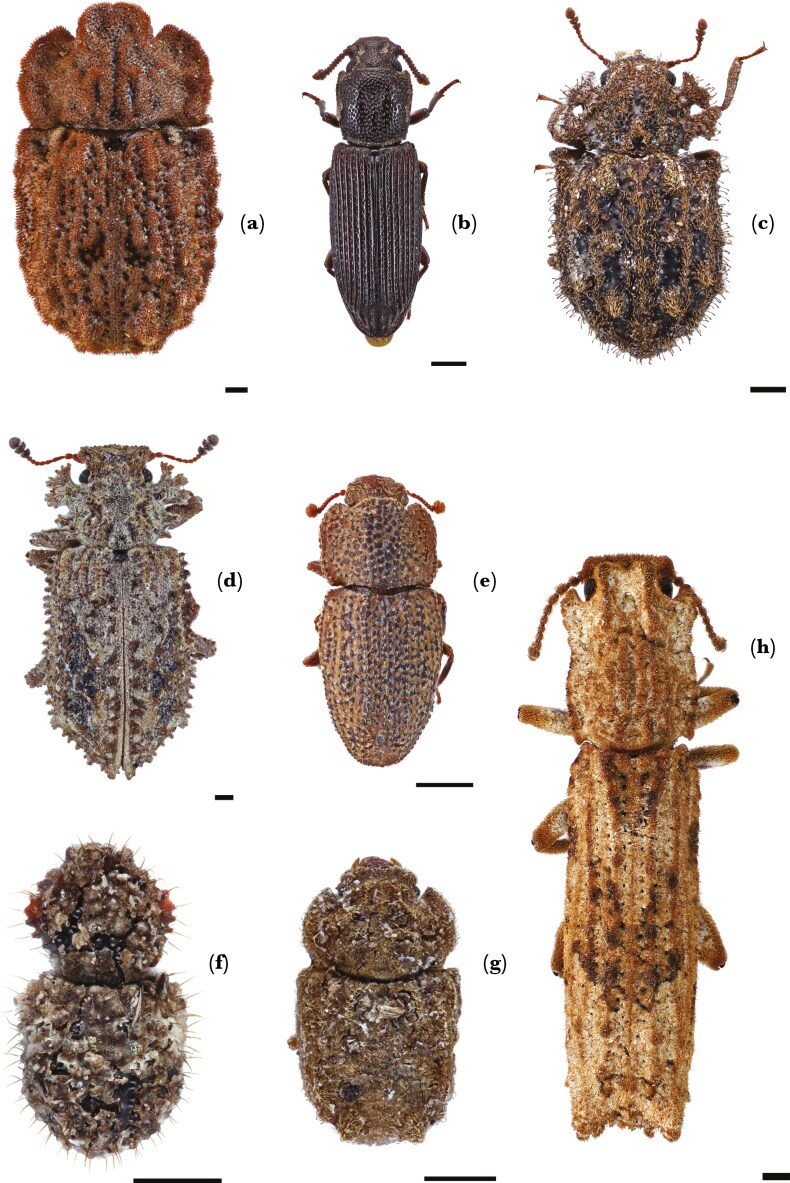
*Zopherid beetle species used for this project. From top left: (a). Pristoderus bakewelli (not cloaked), (b). Pycnomerus sp.1 (not cloaked), (c). Pristoderus insignis (not cloaked), (d). Tarphiomimus indentatus (partially cloaked), (e). Chorasus sp.1 (partially cloaked), (f). Epistranus c. f. lawsoni (fully cloaked), (g). Protarphius sp. 1 (fully cloaked), (h). Rytinotus squamulosus* (fully cloaked). Scale bars = 0.5mm.

To test the predictions that passive debris cloaking reduces detection and attack by predatory ants, the degree of passive debris cloaking was classified for each prey specimen as one of three categories. (1) Not cloaked: cuticle on dorsal surface mostly free of secretions and/or debris except for small deposits within punctures or at the base of some setae. (2) Partially cloaked: significant secretion buildup, but with parts of the cuticle and/or setae exposed. (3) Fully cloaked: the entire cuticle on the dorsal surface (not including appendages and sensory setae protruding from cuticle) fully covered with secretions and / or debris. Although there is substantial variation in the degree of encrustation between species, only species that could easily be placed into one of the three categories were used for experimentation (Fig. 1a-h). To determine how ants interacted with beetles compared to pieces of natural bark debris (taken from woody debris sifting extractions that contained zopherids), we included two prey control groups: large prey control (bark size: length 6 to 10 mm × width 2 to 5 mm) and small prey control (bark size: length 2 to 4 mm × width 1 to 2 mm). Each prey specimen and ant colony were given unique identifiers to account for individual differences in responses. These are referred to as “prey reference” and “colony code” respectively. To determine whether size had a significant effect, we calculated prey length and width as means for each species to include in statistical analysis.

To test the hypothesis that detection by predatory ants is less likely on natural backgrounds, we used three background treatment types: (1) bare arena: foraging area clean of dirt and debris. (2) camouflage arena: prey specimen was placed onto a piece of bark in the foraging area for the duration of the experiment, this treatment represents a natural background for zopherids. (3) debris arena: foraging area was scattered with woody debris particles. This treatment was to assess whether having natural environmental materials in close proximity to zopherid prey, but not in direct contact, could facilitate non-visual camouflage. Each individual beetle was exposed to every treatment type in each of the four ant colonies and experimental order was chosen at random (3 treatments x 4 ant colonies = 12 replicates per beetle).

#### Experimental procedure.

All experiments were scored by the first author. Data were collected either in real time using a red head light, or via infrared recording using a Sony FDR AX40 camcorder and scored at a later date. The camcorder was attached to a Manfrotto tripod (model: MT190XPRO3 with BHQ2 ball head) set up to point the camera directly down at the colony. Experiments commenced 1 h after the start of the dark cycle in a controlled temperature cabinet (9:00 PM at the earliest) as this is when both ant species were at their most active. Experiments were conducted in the dark with only a red LED head lamp for light. Prior to experiments, food was removed from artificial nests and the foraging area was cleaned using a clean paper towel and cotton buds. Experiments were conducted at ambient laboratory temperature (iButton data showed a temperature range from 19 °C to 22 °C with 55% – 60% humidity) and ants were given 10 min acclimation before prey specimens were introduced. Experiments were conducted in the foraging area of each separate artificial ants nest. A single prey was placed in the foraging area of artificial nests using either a paint brush or forceps, with great care taken to ensure beetles were only handled in such a way that oils could not be transferred that could affect cuticle olfaction. They were given 2 min acclimation within an upside-down plastic vial acting as a protective barrier from the ants. The experiment began once the vial was removed and lasted 10 min. Interactions were scored as one of four interaction types, modified from Finlayson, Alyokhin & Porter (Finlayson Alyokhin and Porter 2009); (1) contact without a reaction: this is classified as physical contact between an ant and the beetle without any visible reaction (passive or aggressive) from the ant, commonly seen as an ant walking directly over the beetle without stopping. (2) prolonged antennation: this occurred when an ant placed its antennae in physical contact with the beetle in addition to physical suspension of locomotion. (3) grasping: an ant clasps its mandibles onto any body part of the beetle. (4) dragging/carrying: when an ant physically moved the prey using its mandibles, either in an attempt to detach an appendage or by carrying the entire beetle.

The exposure of beetles to two ant species consecutively could result in changes in their cuticular chemicals during multiple trials, which may influence predator behavior. To address this, the replicate number for each beetle (1 to 12) was recorded and incorporated as a random factor in statistical analyses. In cases where the beetle crawled off the bark in the “camouflage” treatment, a paintbrush was used to put it back. This usually resulted in zopherid beetles suspending movement and retracting appendages, a defensive posture. To avoid any influence of this retracted posture, scoring was suspended immediately after this, and only recommenced once the beetle was dorsally oriented with their antennae released. This was to avoid any scoring bias that may occur as the result of antennal contact with the beetle’s ventral side, which has considerably less debris than the dorsal surface.

#### Statistical analysis – detection.

To determine whether the degree of debris cloaking had an effect on detection, a generalized linear mixed effects model (GLMM) with a binomial distribution and logit link function was used. Many ant behavioral studies focus on aggression, and prolonged antennation is classed as a passive behavior, and is coded as such in related studies (Finlayson et al. 2009; Garnas et al. 2014; Nelson and Mooney 2022). Olfactory camouflage relates more strongly to avoiding detection entirely rather than minimizing aggression, so for the purpose of measuring detection, “prolonged antennation” is considered a reactive behavior as it represented a clear inspection of the beetle beyond random contact with no reaction. Data were transformed to binary, with “contact without reaction” as 0 and all other beetle/ant interactions (“prolonged antennation,” “grasping,” and “dragging/carrying”) as 1. As each prey species had a consistent value for coverage, “species” was nested within ‘cloaking’. Model selection was determined using model reduction techniques implemented using *lme4* package in R (Bates et al. 2009; R Core Team 2023), initially including all factors mentioned above until a model of best fit was determined. This involved including all factors initially, removing one at a time until factors that significantly influenced the AIC value were determined (a change of 2 or more). Prey size (width mm and length mm), and prey replicate number (1 to 12 for each individual beetle) were found to have no effect so were removed from the model. The final model included predictor variables = predator + treatment * (cloaking/prey), and response variable = interaction, with random effect terms for prey reference (χ^2^ = 623.12, p < 0.0001) and colony code (χ^2^ = 60.406, p < 0.0001), with a binomial error distribution and bobyqa optimizer increased to 2 million function evaluations to allow for model convergence. The *DHARMa* package (Hartig and Lohse 2022) was used to check for overdispersion, outliers and uniformity by simulating residuals from the fitted model. Pseudo R squared values were used to check explained variance of the full model against a null model using *MuMIn* package (Barton 2023). Differences amongst ants, prey, and treatments were determined by post hoc comparisons on the log odds scale using Bonferroni corrections from the *emmeans* package (Lenth 2023) in R. Results are back transformed to the response scale.

#### Statistical analysis – aggression.

In cases where prolonged antennation was observed, it wasn’t always accompanied by aggressive predatory reactions, as it is not typically regarded as an act of aggression (Finlayson et al. 2009; Garnas et al. 2014; Nelson and Mooney 2022). For this reason, it is possible that ant predators may have been misidentifying prey individuals as something inedible, suggesting masquerade (Skelhorn et al. 2010b). To determine how often detection of prey led to predation attempts, contact without a visible reaction was excluded from the data and prolonged antennation was re-coded as 0 for binary analysis. Aggressive interactions (grasping and carrying/dragging) were coded as 1. Controls were also excluded due to the very small number of times ants showed signs of aggression towards bark controls (total data points after exclusions n = 9507). A GLMM was run with identical parameters to the detection analysis. Post hoc comparisons were performed identical to above.

## Results

### Detection

We found a significant effect of background type (χ^2^ = 992.108, Df = 2, p < 0.0001) and degree of passive debris cloaking (χ^2^ = 204.310, Df = 3, p < 0.0001) on the probability of detection by predatory ants. Pairwise comparisons between the three background types and between the three cloaking categories and controls are presented in Tables 2 and 3.

**Table 2.** Pairwise comparisons for the rate of detection and attack between (A) the three different backgrounds and (B) the degree of cloaking for zopherids.

Comparison	Detection model			Aggression model		
	Odds ratio	z	p	Odds ratio	z	p
**(A) Backgrounds**						
Bark / Debris	0.698	−5.102	<0.0001	0.604	−2.274	0.069
Bare / Bark	3.52	21.072	<0.0001	2.54	4.618	<0.0001
Bare / Debris	2.360	13.866	<0.0001	1.5	3.413	0.0019
**(B) Cloaking**						
Full / Uncloaked	0.386	−8.249	<0.0001	0.662	−3.260	0.0033
Full / Partial	0.734	−1.173	0.5007	2.016	2.587	0.029
Uncloaked / Partial	1.899	3.559	0.0022	3.047	4.163	0.0001
Control / Full	0.393	−6.632	<0.0001	-	-	-
Control / Partial	0.288	−6.326	<0.0001	-	-	-
Control / Uncloaked	0.152	−13.233	<0.0001	-	-	-

**Table 3.** Pairwise comparisons for the rate of detection and attack between the three different backgrounds assessed separately for each cloaking category and for controls.

Backgrounds	Cloaking amount	Detection model			Aggression model		
		Odds ratio	z	p	Odds ratio	z	p
Bare / Bark	Full	3.65	18.735	<0.0001	1.85	3.968	0.0002
	Partial	2.83	6.753	<0.0001	6.46	3.259	0.0034
	Uncloaked	4.329	21.369	<0.0001	1.37	2.589	0.0289
	Controls	3.421	2.391	<0.0001	-	-	-
Bare / Debris	Full	2.667	13.813	<0.0001	1.211	1.304	0.3928
	Partial	2.65	6.615	<0.0001	2.509	2.755	0.0176
	Uncloaked	2.288	13.677	<0.0001	1.190	1.864	0.1491
	Controls	1.322	2.394	0.05	-	-	-
Bark / Debris	Full	0.731	−3.916	0.0003	0.655	−2.236	0.0653
	Partial	0.95	−0.427	1	0.388	−1.514	0.2841
	Uncloaked	0.528	−9.558	<0.0001	0.866	−1.133	0.4936
	Controls	0.387	−6.065	<0.0001	-	-	-

**Fig. 2.**
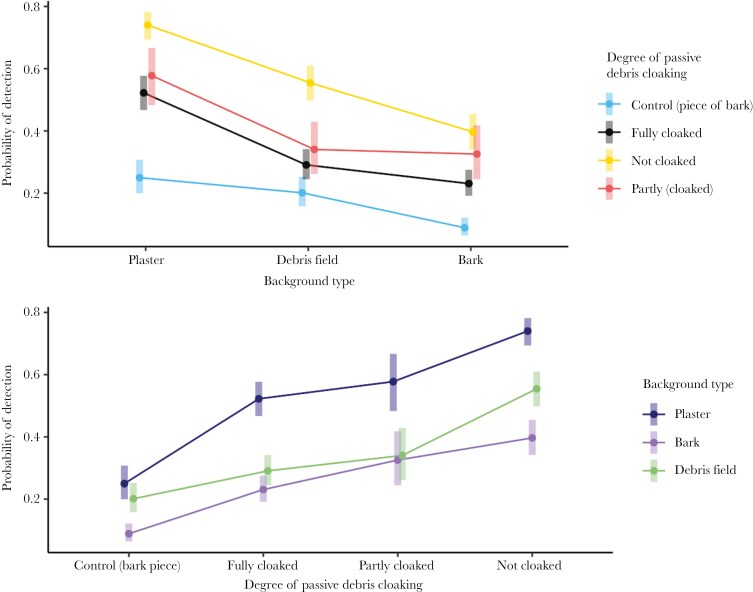
(a) Estimated marginal means from generalized linear mixed effects logistic regression model (binary distribution with logit link function, bobyqa optimizer set to 2 million function evaluations) displaying probabilities of detection of zopherids on differing background types (Yellow = Control group, Dark green = prey with full debris coverage, Light green = prey with partial debris coverage, Black = prey without debris coverage). (b) Estimated marginal means from generalized linear mixed effects logistic regression model (binary distribution with logit link function, bobyqa optimizer set to 2 million function evaluations) displaying probabilities of detection for differing degrees of passive debris cloaking (Blue = plaster control treatment type, Purple = bark camouflage treatment type, Red = debris treatment type).

We found that predatory ants detected zopherids most frequently on bare backgrounds (55.2% ± 2.4%), followed by debris backgrounds (33.7% ± 2.2%) and least frequently on bark backgrounds (23.7% ± 1.9%) (Table 1, Fig. 2a). Pairwise comparisons showed predatory ants were more likely to detect zopherid prey from all cloaking categories on bare backgrounds than on debris or bark (Tables 2 and 3). As we predicted, fully cloaked zopherids were detected the least frequently compared to other cuticle types (Table 1, Fig. 2b). Pairwise comparisons showed fully cloaked and partially cloaked zopherids were significantly less likely to be detected than uncloaked species (Fully cloaked: z = −8.249, p < 0.0001; Partially cloaked: z = 3.559, p = 0.0022), however the difference between detection of fully cloaked and partially cloaked species was not significant (z = −1.173, p = 0.5007) (Table 2).

**Table 1.** Proportion of beetles detected and if detected, attacked by predatory ants according to different background types and degree of cloaking with 95% confidence intervals (GLMM with binomial distribution).

Variable	Detection model	Aggression model
	Proportion (95% CI)	Proportion (95% CI)
**Backgrounds:**		
Bare	0.52 (0.474, 0.569)	0.20 (0.146, 0.274)
Debris	0.34 (0.295, 0.381)	0.14 (0.097, 0.204)
Bark	0.24 (0.202, 0.275)	0.09 (0.055, 0.146)
**Cloaking amount:**		
Fully cloaked	0.34 (0.292, 0.389)	0.15 (0.106, 0.211)
Partially cloaked	0.41 (0.331, 0.496)	0.08 (0.0455, 0.140)
Uncloaked	0.57 (0.516, 0.623)	0.21 (0.153, 0.286)
Controls	0.17 (0.133, 0.208)	-

Pairwise comparisons revealed three species that showed no significant difference to the bark controls. These were *Tarphiomimus indentatus* (partially cloaked) and *Protarphius* sp. 1 (fully cloaked) compared to the large bark control (*T. indentatus:* z = −1.872, p = 1, *Protarphius* sp. 1: z = −1.725, p = 1), and *Epistranus* c. f. *lawsoni* compared to both the large and small bark controls (large bark control: z = 0.619, p = 1; small bark control: z = −3.179, p = 0.0665). Full species detection proportions and pairwise comparisons can be found in electronic supplemental material (Tables S1 and S2). Lastly, there was a difference between the detection rates of both predatory ant species (χ^2^ = 15.779, Df = 1, p < 0.0001). *Amblyopone australis* was less likely to detect any zopherid than *Austroponera castanea* (odds = 0.547, z = −3.972, p = 0.0001).

### Aggression

There was a significant effect of background type (χ^2^ = 32.5774, Df = 2, p < 0.0001), and degree of passive debris cloaking (χ^2^ = 30.9698, Df = 2, p < 0.0001) on the probability of attack from predatory ants, if detected. Pairwise comparisons are in Tables 2 and 3.

The aggression model showed that when predatory ants detected zopherids, they attacked them most frequently on bare backgrounds (20.3% ± 3.3%), followed by debris backgrounds (14.2% ± 2.7%), and least frequently on bark backgrounds (9.1% ± 2.3%). However, the difference between bare and debris was only significant for partially cloaked species (Table 3), and the difference between bark and debris was marginally non-significant (z = −2.274, p = 0.069) (Table 2).

**Fig. 3.**
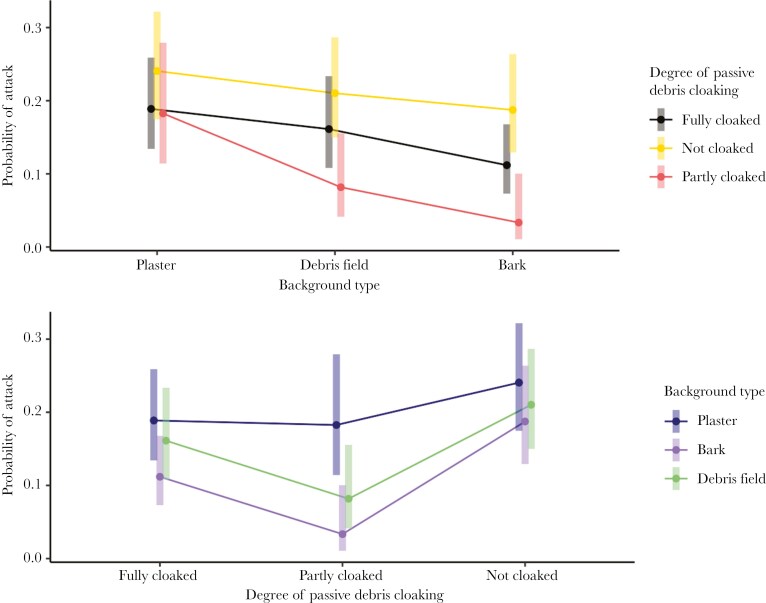
(a) Estimated marginal means from generalized linear mixed effects logistic regression model (binary distribution with logit link function, bobyqa optimizer set to 2 million function evaluations) displaying probabilities of attack, if detected, on zopherids on different treatment types (Red = prey without debris coverage, Black = prey with full debris coverage, Yellow = prey with partial debris coverage). (b) Estimated marginal means from generalized linear mixed effects logistic regression model (binary distribution with logit link function, bobyqa optimizer set to 2 million function evaluations) displaying probabilities of attack, if detected, on zopherids with differing levels of debris coverage (Light blue = plaster control treatment type, Medium blue = bark camouflage treatment type, Dark blue = debris treatment type).

As expected, fully and partially cloaked zopherids were less likely to be attacked if they were detected than uncloaked species (Table 2, Fig. 3b). However, we found that if detected, fully cloaked prey were more likely to be attacked than partially cloaked prey (z = 2.587, p = 0.029). The probability of attack after detection did not differ between ant predator species *Amblyopone australis* and *Austroponera castanea* (z = 0.241, p = 0.8093).

## Discussion

By exposing zopherid beetles with varying degrees of passive debris cloaking to predatory ants, we found that passive debris cloaking dramatically reduces the probability of detection by ants. We also found that zopherids with passive debris cloaking were less likely to be attacked if detected, compared to species with no cuticular debris. Furthermore, we found that partial passive debris cloaking was superior at reducing aggression to full passive debris cloaking, however the likelihood of detection did not differ between full and partial cloaking amounts. We also found that detection and attack were less likely when zopherids were on backgrounds of bark or debris. Our results suggest passive debris cloaking is an effective form of non-visual camouflage against olfactory driven predators.

These results are similar to those found for decorating behaviors in other arthropods (Wicksten 1980; Cruz-Rivera 2001; Brandt and Mahsberg 2002; Hultgren and Stachowicz 2009). Decorating is similar to passive debris cloaking but involves the deliberate selection and attachment of debris, as described in Ruxton and Stevens (2015). Most research has focused on the physical protection and visual camouflage that decorating provides (Eisner et al. 1978; Olmstead and Denno 1993; Brandt and Mahsberg 2002; Jackson and Pollard 2007). However, there is some experimental evidence supporting its function as non-visual camouflage. The trash packet (a form of decorating) of the larval form of *Plagiometriona clavata* (folivorous tortoise beetle) was found to be a successful chemical defence, where chemical alteration of the trash packet increased predation by ants (Vencl et al. 1999). Similar evidence of chemosensory concealment against ants was found in laboratory experiments involving decorating wax on *Chrysopa slossonae* larvae (green lacewing) (Eisner et al. 1978) and the dust coating in nymphs of *Paredocla* and *Acanthaspis* spp. (West African assassin bugs) (Brandt and Mahsberg 2002). Similarly, the predatory nymphs of *Salyavata variegata* (assassin bug from Costa Rica) conceal their scent by using part of the wall from a termite nest to avoid detection from its prey (McMahan 1982). While these examples demonstrate the chemical benefits of using environmental debris for concealment, they are all from the perspective of the predator. By looking at the use of environmental debris in prey species, we were able to additionally confirm it lowers the likelihood of detection and attack, serving an antipredator function in zopherid beetles. It is worth noting that olfactory reliant predators have other senses that may be exploited with passive debris cloaking. The antennae of ants have mechanical, hygro and thermal sensilla in addition to chemoreceptors (Ramirez-Esquivel et al. 2014). Therefore, while passive debris cloaking appears to be an effective defence against predatory ants, we cannot say for certain whether it is entirely chemical. It is possible that cloaked zopherids are benefitting from multiple properties of the environmental debris covering their cuticles, and in turn exploiting multiple senses of predatory ants.

Background matching is a term commonly used in visual camouflage research that can be easily applied to non-visual cues. In this case an animal might be matching non-visual properties to those of its background (Cuthill et al. 2005). For example, the least killifish *Heterandria formosa* matches visual properties of its habitat to conceal itself (Kjernsmo and Merilaita 2012), and the coral feeding reef fish *Oxymonacanthus longirostris* matches the chemical components of its habitat. Although these camouflage types differ in the predatory senses they exploit, they both match elements of their backgrounds to reduce detection. Passive debris cloaking in zopherid beetles likely functions similarly by matching the cloak’s properties with the debris of the surrounding habitat.

In addition to reducing detection from predators, passive debris cloaking also significantly reduces the probability of attack after detection compared to uncloaked species, an effect which was lowest when combined with natural bark backgrounds. This is likely due to difficulties identifying a cloaked zopherid from the surrounding bark and woody debris, potentially by increasing background noise, an effect also found in visual camouflage. In a study using humans and virtual targets, it was found that camouflaged targets were more difficult to identify when surrounded with similar background objects (Hall et al. 2013). It is possible something similar is occurring here. Predatory ants may struggle to identify a cloaked zopherid when it’s surrounded with bark and debris particles. These results also imply passive debris cloaking has the potential to disrupt multiple stages of the predation sequence (detection and identification), at least in its most extreme cases. Since predatory ants are less likely to attack a zopherid after they’ve detected it when it’s cloaked, it suggests they fail to identify it as a potential food source and are mistaking cloaked zopherids for naturally occurring debris particles. This can be considered a disruption to the identification phase of predation, characteristic of masquerade, where a predator may successfully detect a prey’s presence, but be unsuccessful in identifying it (Skelhorn et al. 2010b). Furthermore, we found that three species of zopherid showed no significant difference in detection to the bark controls (*Tarphiomimus indentatus, Protarphius* sp. 1, and *Epistranus* c. f. *lawsoni*). This shows that the ants were stopping to antennate these species as frequently as they would naturally occurring debris particles. This evidence seems to support passive debris cloaking as a form of non-visual masquerade. Unfortunately, there is limited empirical evidence for masquerade generally, as it’s difficult to confirm experimentally, despite its wide acceptance in theory. The few examples include masquerade in twig mimicking caterpillars (Skelhorn et al. 2010a; Skelhorn and Ruxton 2011, 2013), and flea and leaf beetles masquerading as their own feeding damage (Konstantinov et al. 2018; Folgar‐Cameán et al. 2021). These examples are unlikely to reflect the prevalence of masquerade in nature, as it is certain to be far more widespread than current literature suggests.

Our results confirm passive debris cloaking is a highly effective form of non-visual camouflage, and suggest it may secondarily function as non-visual masquerade. We hope future research will shed light on the specific sensory modalities involved here. Specifically, it would be useful to determine whether passive debris cloaking is exploiting chemical, tactile, or a combination of sensory cues. Additionally, there are several examples of debris cloaking taxa that are also found in edaphic habitats, including several species of mites, harvestmen, flat bugs, and other beetles (Mahunka 1994; Olszanowski 1996; Leschen et al. 2005, 2016; Łochyńska 2008a, 2008b; Bromberek and Olszanowski 2012; Wolff and Gorb 2016; Porto and Pérez-González 2020; Larivière and Larochelle 2022). Therefore, it would be valuable to understand the function of passive debris cloaking in some of these other taxa. Future research may reveal passive debris cloaking as a widespread defence against non-visual hunters that dominate low light habitats.

## Supplementary Material

araf064_suppl_Supplementary_Materials

araf064_suppl_Supplementary_Data

## Data Availability

Data available at Dyrad Digital Repository: https://doi.org/10.5061/dryad.v15dv427v. Detailed collection, husbandry, and locality information can be found in Supplementary material.
