# Supplementary material for: Passive debris cloaking in beetles provides non-visual camouflage against predatory ants
Source: Behav Ecol. 2025 Jul 28;36(4):araf064. doi: 10.1093/beheco/araf064 (PMC12302502; doi:10.1093/beheco/araf064)
Supplement: araf064_suppl_Supplementary_Materials [file araf064_suppl_supplementary_materials.zip › araf064_suppl_Supplementary_Materials/Supplementary material.docx]

**Supplementary material for *“Passive debris cloaking provides non-visual camouflage against predatory ants”***

**Collection and husbandry**

**Field collection of ants**

Ant colonies were extracted from nests found inside large logs and punga fern trunks found on the forest floor at Hunua Ranges Regional Park (37°06'32.3"S 175°08'46.6"E). Hand tools were used where necessary to access deeper chambers with brood. Contents of nests were transported back to the laboratory in pillowcases where they were extracted using a field aspirator to eliminate soil prior to being placed in artificial nests. All colonies were placed in artificial nests within 24 hours of field extraction. Four separate ant colonies were used for experiments, 2 x *Austroponera castanea* a native species*,*; and 2 x *Amblyopone australis* which is generally considered an exotic species, introduced from Australia (Ward, 2009, Ward, 2005), however it’s status is still in dispute due to slight morphological differences in the New Zealand fauna and fossil evidence of similar specimens from the early miocene (Kaulfuss and Dlussky, 2015, Kaulfuss et al., 2014, Taylor, 1978). All colonies had brood present at time of extraction to ensure worker behaviour was as close to natural as possible.

**Housing of laboratory ant colonies**

All ant colonies were kept in 2L rectangle Sistema^®^ plastic containers with a plaster of Paris mixture (1:1 plaster/water by volume) with one tablespoon of activated charcoal to increase absorption and decrease mould growth. When plaster was still wet, foam circles were placed into the mixture in half of the container to create artificial nest moulds. Clear acrylic panels (100mmx140mmx2mm) were then pressed over the foam circles until the acrylic was flush with the plaster surface to create an enclosed “nest”. Once dry, the foam was removed and an entrance was carved from the plaster to allow ants to enter the chambers while the acrylic sheet shielded the nest area. Red cellophane was glued (PVA) onto the acrylic sheet to produce continuous darkness within the chambers, while allowing easy observation of the ants inside. The second half of container was left empty to act as the workers’ ‘foraging zone’. This is where the food was placed, and also where the experiments were conducted. A talc/ethanol mixture at a concentration of 20% was brushed onto the sides of container to prevent ant escape (Ning et al., 2019). Several holes were drilled into the lids for air circulation and closed when colonies weren’t in use for experiments. Large pieces of chiffon fabric were placed over the container and clipped in place with the lid as an added precaution to prevent escape. Prior to introducing ants to the completed artificial nests, the plaster was left for at least 2 days to completely dry, then soaked with RO water for 10 minutes to rehydrate and introduce humidity, excess water was soaked up with paper towel.

Containers were kept in a controlled temperature cabinet set to 14:10 L/D cycle and 20°C/18°C temperature cycle. Temperature conditions were lowered slightly from MetService (MetService, 2020) historical data (22°C-23°C average from December-January) taking into account nest insulation and cooler ambient temperatures of the ants usual habitats (forests under canopy cover). All ant colonies were acclimated for a minimum of two weeks in controlled temperature conditions prior to being used in experiments (Wittman et al., 2018, Rowles and O’Dowd, 2007).

**Feeding and maintenance of colonies**

Ants were initially fed two hours after introduction into the artificial nests to allow workers time to place brood into the nest chambers and clear the foraging area, and then three times a week for the following six weeks, after which feeding was reduced to once a week to induce mild starvation and increase behavioural responses. Ants were fed a combination of sugar water (1:1 ratio by weight) and a protein source in the form of live meal worms. Sugar water was soaked into a small piece of cotton wool (roughly the size of a pea) placed on a 3cmx3cm piece of aluminium foil set in foraging area of each ant colony. 1 large or 2 small meal worms were used each feed, old meal worm cases were removed during the following feeding event to prevent mould growth. During each feed, plaster was rehydrated with RO water and additionally when required to maintain humidity.

**Field collection of zopherids**

Zopherids used in experiments were collected on 12 separate occasions throughout October 2022 to March 2023, across 4 locations in and around Auckland: Hunua Ranges Regional Park, Kepa Bush Reserve, Wattle Bay Reserve, Maungatautari Sanctuary Mountain, in addition to Mt. Arthur and Harwoods Hole near Nelson on the South Island. Due to the saproxylic and mostly nocturnal nature of Zopheridae beetles, specialised collecting techniques were required to find them in abundance. Five techniques were used in total: 1) sifting, the majority of specimens used were collected with this technique; a litter sifter with 1 cm x 1 cm mesh sieve was used to sift woody debris and twigs, usually with fungus. The material collected was then taken back to the laboratory and put in a Berlese funnel until material was completely desiccated. This took anywhere from 2 to 6 weeks depending on the moisture content of the sample. 2) beat sheets were used to target species that live above the litter layer in shrubs showing signs of decay such as *Tarphiomimus indentatus,* 3) hand collecting was undertaken during the day, which only included a single specimen 4) at night with a standard head torch, and 5) at night with a UV hand torch, specifically to collect *Rytinotus squamulosus.* This is by far the easiest technique to collect this species due to their green biofluorescence under UV light.

**Feeding and housing of Zopheridae beetles**

Zopheridae beetles were housed in specimen containers at room temperature, with a mesh window for airflow and plaster of Paris x activated charcoal base (same ratio as artificial nests). They were fed dried shitake mushrooms and plaster was kept damp with RO water. Mushrooms were changed a minimum of 3 times a week to prevent mould growth. In cases where mould growth occurred, zopherids were transferred into clean containers with fresh plaster x charcoal base. After extraction (from Berlese funnels or field if hand collecting) Zopherids were kept in artificial housing prior to experimental use for a maximum of 2 weeks. Species used in experimental trials were: *Pristoderus bakewelli, Pristoderus insignis, Tarphiomimus indentatus, Epistranus* cf. *lawsoni, Protarphius* sp. 1*, Chorasus* sp. 1*, Pycnomerus* sp*.* 1*,* and *Rytinotus squamulosus* (n=110).

**Note on attempts to remove debris**

Several attempts failed to remove the debris from different zopherid species - ranging in size from 1.5 mm to 5.0 mm. Morphological studies of preserved material led to some ability to remove encrustations affixed by flaking them off carefully with minuten pins and micro tweezers alongside the use of solvents (ethanol, acetone, degreasers). The process was slow and meticulous and still unfortunately resulted in the loss of scales and distortion of the cuticle. Additional failed attempts to remove encrustations from preserved specimens included up to 24 hours in a sonicator. Pilot attempts to remove the debris cloaks from live specimens was even more challenging. Physical removal of debris resulted in the loss of legs or death. This therefore made an experimental modification of debris cloaking impossible. We then decided to increase the number of species used, which varied in their degree of debris cloaking, to employ a powerful comparative approach for assessing predatory responses to variation in passive debris cloaking.

**Results – individual species**

**Table S1.** Estimated marginal means comparisons for detection probabilities between large and small bark controls, and zopherid species. Model output from generalized linear mixed model with binomial distribution. Bonferroni corrections are applied to p values.

| **Comparisons** | **Coverage** | **Odds ratio** | **z** | **p** |
| --- | --- | --- | --- | --- |
| **Large control:** |  |  |  |  |
| ***Epistranus* c. f. *lawsoni*** | **Fully cloaked** | **1.141** | **0.619** | **1** |
| ***Protarphius* sp. 1** | **Fully cloaked** | **0.709** | **-1.725** | **1** |
| *Rytinotus squamulosus* | Fully cloaked | 0.266 | -6.105 | < 0.0001 |
| *Pristoderus bakewelli* | Nude | 0.179 | -8.16 | < 0.0001 |
| *Pycnomerus* sp.1 | Nude | 0.381 | -4.233 | 0.001 |
| *Pristoderus insignis* | Nude | 0.182 | -8.59 | < 0.0001 |
| *Chorasus* sp.1 | Partially cloaked | 0.332 | -4.302 | 0.0008 |
| ***Tarphiomimus indentatus*** | Partially cloaked | 0.582 | -1.872 | **1** |
| **Small control:** |  |  |  |  |
| ***Epistranus* c. f. *lawsoni*** | **Fully cloaked** | **0.490** | **-3.179** | **0.0665** |
| *Protarphius* sp.1 | Fully cloaked | 0.305 | -5.621 | < 0.0001 |
| *Rytinotus squamulosus* | Fully cloaked | 0.114 | -9.501 | < 0.0001 |
| *Pristoderus bakewelli* | Nude | 0.077 | -11.53 | < 0.0001 |
| *Pycnomerus* sp.1 | Nude | 0.164 | -7.583 | < 0.0001 |
| *Pristoderus insignis* | Nude | 0.078 | -12.092 | <0.0001 |
| *Chorasus* sp.1 | Partially cloaked | 0.143 | -7.311 | <0.0001 |
| *Tarphiomimus indentatus* | Partially cloaked | 0.25 | -4.652 | 0.00014 |

**Table S2:** Zopherid species proportions for generalized linear mixed effects models with binomial distributions for the likelihood of detection and attack from predatory ants with 95% confidence intervals.

| **Species** | **Cloaking amount** | **Detection** | | **Attack** | |
| --- | --- | --- | --- | --- | --- |
|  |  | **Proportions (%)** | **95% CI** | **Proportions (%)** | **95% CI** |
| *Epistranus* c. f. *lawsoni* | Fully cloaked | 0.21 | 0.16, 0.27 | 0.18 | 0.11, 0.27 |
| *Protarphius* sp.1 | Fully cloaked | 0.3 | 0.25, 0.36 | 0.21 | 0.14, 0.29 |
| *Rytinotus squamulosus* | Fully cloaked | 0.54 | 0.45, 0.62 | 0.09 | 0.06, 0.14 |
| *Pristoderus bakewelli* | Uncloaked | 0.63 | 0.56, 0.7 | 0.33 | 0.24, 0.43 |
| *Pycnomerus* sp.1 | Uncloaked | 0.45 | 0.36, 0.53 | 0.08 | 0.05, 0.13 |
| *Pristoderus insignis* | Uncloaked | 0.63 | 0.56, 0.69 | 0.3 | 0.22, 0.4 |
| *Chorasus* sp.1 | Partially cloaked | 0.48 | 0.38, 0.59 | 0.13 | 0.08, 0.22 |
| *Tarphiomimus indentatus* | Partially cloaked | 0.35 | 0.24, 0.47 | 0.05 | 0.02, 0.11 |
